# A novel rat model of interbody fusion based on anterior lumbar corpectomy and fusion (ALCF)

**DOI:** 10.1186/s12891-021-04822-4

**Published:** 2021-11-18

**Authors:** Yu Kang, Chao Liu, Ming Wang, Cheng Wang, Yi-Guo Yan, Wen-Jun Wang

**Affiliations:** 1 The First Affiliated Hospital, Department of Spine Surgery, Hengyang Medical School, University of South China, Hengyang, 421001 Hunan China; 2 Department of Orthopedicsity, The First Affiliated Hospital of Anhui Medical University, Hefei, 230000 Anhui China

**Keywords:** Animal model, Interbody fusion, Micro-CT, Histology

## Abstract

**Background:**

Rats have been widely used as experimental animals when performing fundamental research because they are economical, rapidly reproducing, and heal quickly. While the rat interbody fusion model has been applied in basic studies, existing rat models generally have shortcomings, such as insufficiently simulating clinical surgery. The purpose of this study was to develop a novel rat model of interbody fusion which more closely represents clinical surgery.

**Methods:**

The internal fixation was designed based on physical measurements of the rats’ lumbar spine. Then, ten rats divided into two groups (A and B) underwent anterior lumbar corpectomy and fusion of the L5 vertebrae. Groups A and B were sacrificed four and 8 weeks post-surgery, respectively. Micro-CT and histological examination were used to evaluate the model. Fusion rate, bone volume fraction (BV/TV), trabecular bone number (Tb.N), trabecular bone thickness (Tb.Th), and the area ratio of newly formed bone (NB) were calculated for quantitative analysis.

**Results:**

Based on the L5 body dimensions of individual rats, 3D-printed titanium cage of the appropriate size were printed. The operations were successfully completed in all ten rats, and X-ray confirmed that internal fixation was good without migration. Micro-CT suggested that fusion rates in group B (100%) were greater than group A (40%, *P* < 0.05). The BV/TV (B: 42.20 ± 10.50 vs. A: 29.02 ± 3.25, *P* < 0.05) and Tb.N (B: 4.66 ± 1.23 vs. A: 1.97 ± 0.40, *P* < 0.05) were greater in group B than A, and the Tb.Th in group B was lower than group A (B: 0.10 ± 0.04 vs. A: 0.15 ± 0.02, *P* < 0.05). Histomorphometry results demonstrated that the area ratio of NB in group B were greater than group A (B: 35.72 ± 12.80 vs. A: 12.36 ± 16.93, *P* < 0.05).

**Conclusion:**

A rat interbody fusion model based on anterior lumbar corpectomy and fusion has successfully been constructed and verified. It could provide a new choice for fundamental research using animal models of spinal fusion.

## Introduction

Spinal fusion has become a routine technique for treating lumbar spine degeneration, cervical spine instability, intervertebral disc injury, and spinal deformity [1]. The operation aims to achieve a solid arthrodesis of spinal segments that can sustain loading while maintaining intervertebral disc space height, preserving foraminal dimensions, and restoring sagittal plane alignment. The first case of instrumented spinal fusion was described by Hadra in 1891 [2]. In 1911, orthopedic surgeons Albee and Hibbs independently reported similar spinal fusion procedures for patients with Pott’s disease [3, 4]. Over the last 50 years, spinal fusion has become the gold standard for treating severe degenerative spinal disorders [5]. At present, a variety of implants and surgical instruments are used in clinical practice to improve the outcomes of spinal fusion surgery; pedicle screw and titanium mesh fixation systems are the most commonly employed. However, pseudoarthrosis (failed fusion) rates are as high as 40% following primary spinal fusion and up to 60% in revision cases, even when performing the gold standard method of grafting bone from the patient’s own iliac crest [6, 7].

To overcome the challenges of spinal fusion, researchers have been exploring effective methods of promoting bone regeneration between vertebral bodies based on animal models [8–10]. Koerner and Ryu used an intertransverse process fusion model to study the effect of rhBMP-2 and A/BMP2 on interbody fusion [8, 9]. These studies utilized a rat posterolateral spinal fusion model, constructed by implanting bone, or bone fusion material, between two adjacent transverse processes. Other researchers have used the rat coccygeal interbody fusion model to study spinal fusion. Okada successfully induced spinal fusion by implanting an allograft bone from donor Sprague-Dawley (SD) rats into the coccygeal interbody space combined with external fixation [10]. Although the intertransverse process fusion model and the interbody fusion model have been widely used in spinal fusion research [11–15], a validated animal model representing the clinical procedure has not yet been developed.

In this study, anatomical dimensions of the lumbar spine in rats were measured and used to design a titanium cage for intervertebral fusion. The titanium cage was implanted into the rats via anterior lumbar corpectomy and then fixed by a miniature titanium plate and two screws. Intervertebral fusion was evaluated by micro-computed tomography (Micro-CT) and histological examinations. The purpose of this study was to develop a novel rat spinal fusion model that reproduced the clinical procedure, and to provide a validated model for subsequent experimental spinal fusion research.

## Materials and methods

### Animals

All animal experiments in this study were approved by the ethics committee at the First Affiliated Hospital of the University of South China (LL20190412121). A total of twenty SD rats (8-weeks-old, male, 200–240 g, Hunan Silaike Jinda Experimental Animal Co. Ltd., Changsha, Hunan, China) were used. Ten rats were dissected for vertebral body measurements. Ten rats were randomly divided into two groups before undergoing the modeling operation. Group A (*n* = 5) were sacrificed 4 weeks after the operation, and group B (n = 5) after 8 weeks.

#### Measurements of the vertebral body

**Fig. 1:**
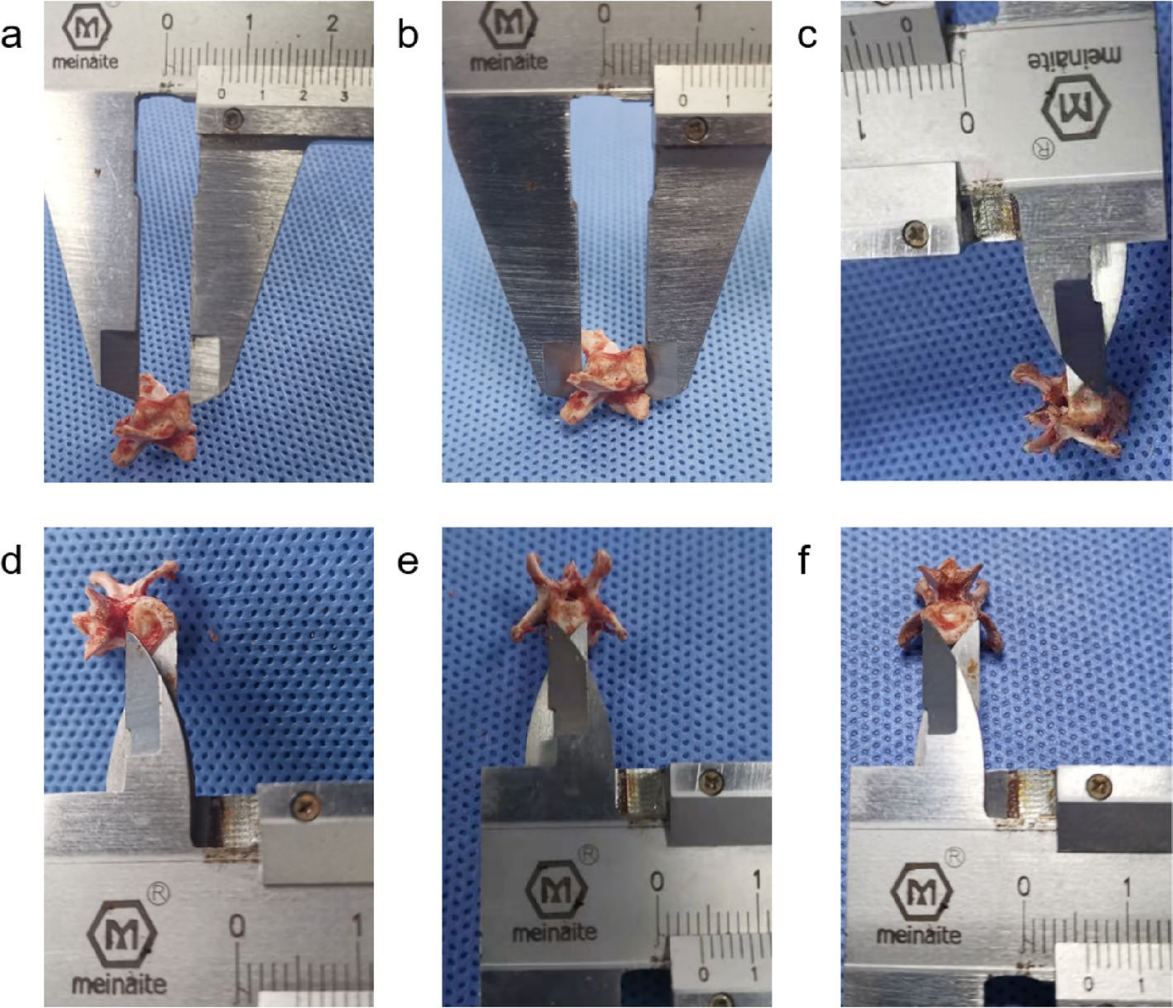
Physical measurement of the L5 vertebral body. **a** Anterior height. **b** Posterior height. **c** AP width of the upper endplate. **d** AP width of the lower endplate. **e** Lateral width of the upper endplate. **f** Lateral width of the lower endplate

Of the six lumbar vertebrae in rats, the 5th lumbar vertebrae (L5) was chosen for subtotal resection. The L5 vertebrae of ten rats were dissected for measurements. A vernier caliper was used to measure the anterior and posterior vertebral body heights, and the anteroposterior (AP) and lateral widths of the upper and lower endplate of L5 (Fig. 1).

### Preparation of the internal fixation

**Fig. 2:**
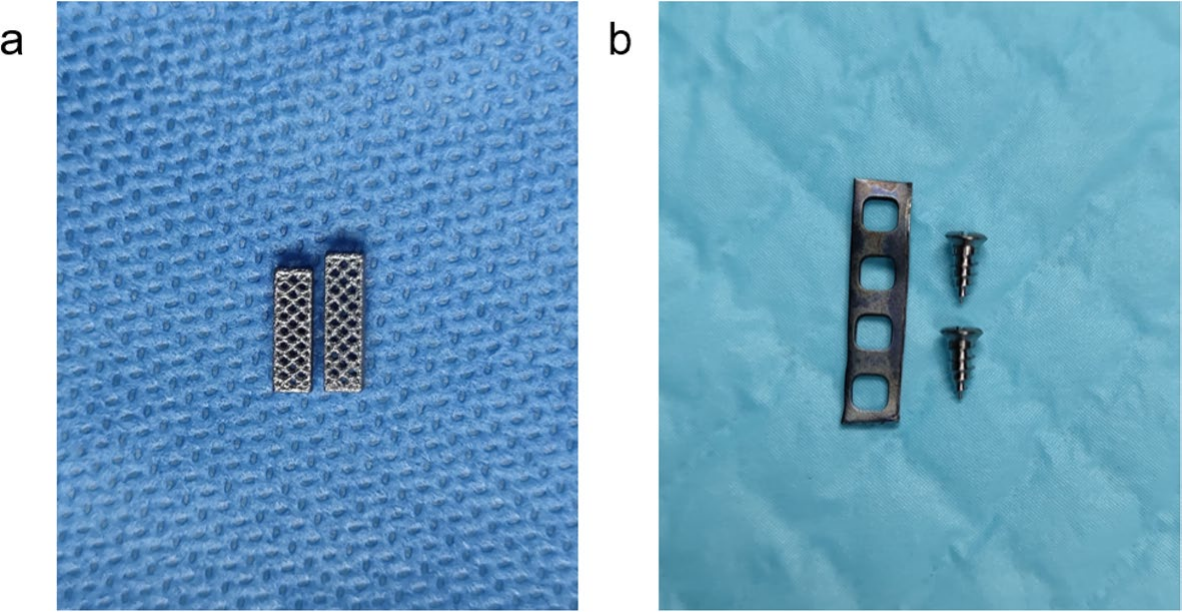
The internal fixation for ALCF. **a** Two size of titanium cage. **b** Titanium plate and screws

The internal fixation was composed of a titanium cage, a titanium plate, and two screws (Fig. 2). The digital model of the titanium cage was constructed with UG (Unigraphics NX10.0) software before being imported into the BuildStar V1.2 software for pre-printing. The titanium cages were printed by selective laser melting (SLM) in a 3D-Printer (Type: FS121M for metal, Farsoon Technologies Co. Ltd., Changsha, Hunan, China). Finally, the products were subjected to post-processing, including de-powdering, separation, de-supporting, polishing, and sandblasting. The titanium plate and screws were supplied by Tianjin Kanger Medical Treatment Apparatus Co., Ltd.

### Surgical procedure

**Fig. 3:**
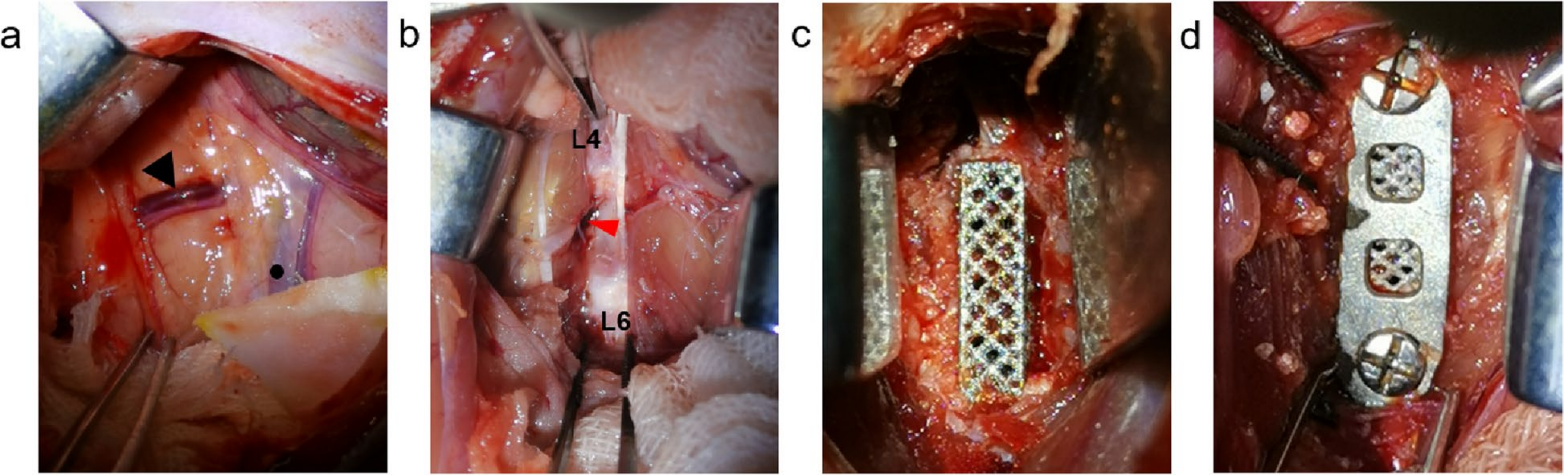
Exposure of the L5 vertebral body and implantation of the inner fixation. **a** Black arrow: ilio-lumbar vessels; black dot: posterior vena cava. **b** The L5 vertebral body and half of the superior and inferior vertebral bodies were fully exposed, red arrow: vertebral vessel. **c** The titanium cage was implanted after corpectomy of L5. **d** The titanium cage was fixed with a titanium plate and screws

The rats were anesthetized with 2% sodium pentobarbital (40 mg/kg, i.p.) [16]. If additional anesthesia was necessary, a further one quarter of the first does of pentobarbital sodium was given. After shaving the abdominal skin and disinfecting with iodine volts, the rats were fixed on the plank in a supine position. To open the peritoneal cavity, a 5-cm long abdominal midline skin incision was made, followed by separation of the subcutaneous tissue to expose the rectus abdominis. Both rectus abdominis muscles were lifted with two pairs of tweezers and a midline incision along the linea alba was made, to ensure the abdominal organs could not be injured. The abdominal organs and intestines were gently placed on saline-soaked gauze to the left and right sides, and the operative field was maintained with two skin retractors. The ilio-lumbar vessels and the posterior vena cava covered by adipose tissue could be seen in the surgeon’s visual field (Fig. 3a).

A blunt vertical dissection of the left lateral muscle tissue was made, caudally to the ilio-lumbar vessels and near the posterior vena cava, exposing the ventral aspect of the spine, as previous described [17]. A C-arm X-ray machine and a metal marker were used to verify the vertebral level (L5) via intraoperative fluoroscopy. Then, the L5 vertebral body and half of the superior and inferior vertebral bodies were fully exposed by removing the muscle tissue attached to the ventral aspect of the spine (Fig. 3b). The ilio-lumbar vessels and the posterior vena cava were protected with saline-soaked gauze and pulled aside by the two skin retractors. A grinding drill was used to grind the L5 vertebral body and the two adjacent discs until the lower and upper endplates of L4 and L6, respectively, could be seen clearly. Saline was continuously instilled into the operative area to remove tissue debris and prevent overheating during the procedure. An absorbent cotton piece was inserted into the bone trough to promote compression hemostasis following the resection. When there was no active bleeding, an appropriately sized titanium cage was implanted into the bone trough, ensuring complete connection with the upper and lower vertebral endplates (Fig. 3c). The titanium cage was then fixed in place by a miniature titanium plate and two screws which were fixed in the L4 and L6 using a screwdriver (Fig. 3d). The wound was washed with a diluted iodine complex and sutured with 5–0 absorbable thread layer by layer. The position of the internal fixation was confirmed by X-Ray immediately after surgery. To prevent infection, 20,000 units of penicillin were injected intramuscularly for three consecutive days after the operation. The rats were euthanized under anesthesia after 4 weeks and 8 weeks, depending on their group allocation. The lumbar spine specimens were harvested and fixed in 4% paraformaldehyde [18].

### Micro-CT

The lumbar spine specimens were scanned ex vivo using micro-CT (Skyscan 1172, Bryker, Belgium) at a resolution of 9 μm/voxel. The micro-CT scan protocol was used at 70 kV, 313 μA, 1 mm aluminium, and 180° rotation with an angular step of 0.7°. Visualization and data reconstruction were conducted using NRecon Reconstruction Software (Micro Photonics Inc. Pennsylvania, USA). The scanning range included the lower endplate of L4 to the upper endplate of L6. The appearance of bony bridging at the interbody space on coronal or sagittal images suggested intervertebral fusion [10]. Measurements of bone volume fraction (BV/TV), trabecular bone number (Tb.N), and trabecular bone thickness (Tb.Th) inside the titanium cage were obtained for quantitative analysis.

### Histologic evaluation

After Micro-CT examination, specimens were washed with flowing water for ten minutes and dehydrated with ethanol at 70, 80, 90, and 100% for 24 h at each step. Dehydrated specimens were then treated with xylene for 24 h to remove the residual ethanol. Specimens were immersed in polymeric solution I (every 100 ml of polymeric solution I consisted of 60 ml methyl methacrylate, 35 ml butyl methacrylate, 4.5 ml methyl benzoate, and 0.5 ml polyethylene glycol) and polymeric solution II (consisting of 100 ml polymerization solution I + 0.4 g/100 ml benzoyl peroxide) for 3 days at 4 °C, successively. Polymer solution I and II hardened the tissues so that their microstructure would not be destroyed by microtome. All specimens were embedded in resin and sagittal sections (200 μm thickness) were stained with methylene blue-acid fuchsin (MB/AF). The histological section images were captured using Pannoramic SCAN (3D Histech, Budapest, Hungary). Image-pro 6.0 software was used for calculating the area ratio of newly formed bone (NB) for quantitative analysis.

### Statistical analysis

All statistical analyses were performed using Graphpad Prism version 9.0 software (GraphPad Software, San Diego, CA, USA). Pearson’s chi-square test was used to compare the fusion rates between the two groups. Unpaired t-tests and nonparametric Wilcoxon rank-sum tests were used depending on whether the data conformed to a normal distribution. The results are presented as mean ± standard deviation. A *p*-value of < 0.05 suggested that the difference was statistically significant.

## Results

### L5 body geometry

The rats had a slender L5 vertebral body (Table 1). The height of its ventral aspect was smaller than the dorsal aspect, and the anteroposterior and lateral widths were similar at the upper endplate level. However, the anteroposterior width was smaller than the lateral width at the level of the lower endplate.

**Table 1:** L5 body geometry

L5	Data (mm)
Anterior height	7.05 ± 0.34
Posterior height	7.99 ± 0.29
AP width of upper endplate	3.99 ± 0.12
AP width of lower endplate	4.16 ± 0.14
Lateral width of upper endplate	4.60 ± 0.14
Lateral width of lower endplate	5.52 ± 0.17

### Dimensions of the internal fixation

Based on the anatomical dimensions of L5 and the thickness of the disc, we designed two sizes of titanium cage that were 9 mm or 10 mm in height and 2.5 mm × 2.5 mm in cross-section. The titanium plates were cut to 14 mm × 3 mm. The screws were 2 mm in diameter and 5 mm in length.

### Outcomes of the modeling operation

**Fig. 4:**
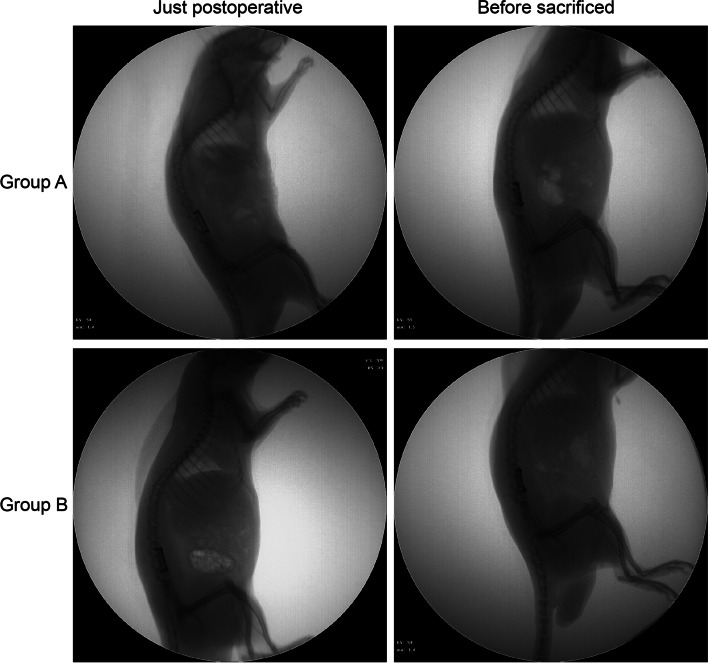
X-ray examination. Postoperative X-ray showed that both ends of the titanium cage were connected with the upper and lower endplates. The posterior vertebral body was intact, and the screws did not intrude on the spinal canal. Before being sacrificed, an X-ray showed that no cage displacement had occurred, and there was no looseness or breakage observed in the screws or plate in either group after their respective postoperative periods

The implants were successfully installed in all rats. Two rats developed wound infections (one in each group) which healed after 1 week. The position of the internal fixation was good, without looseness or breakage, in both groups after their respective postoperative periods (Fig. 4).

### Micro-CT

**Fig. 5:**
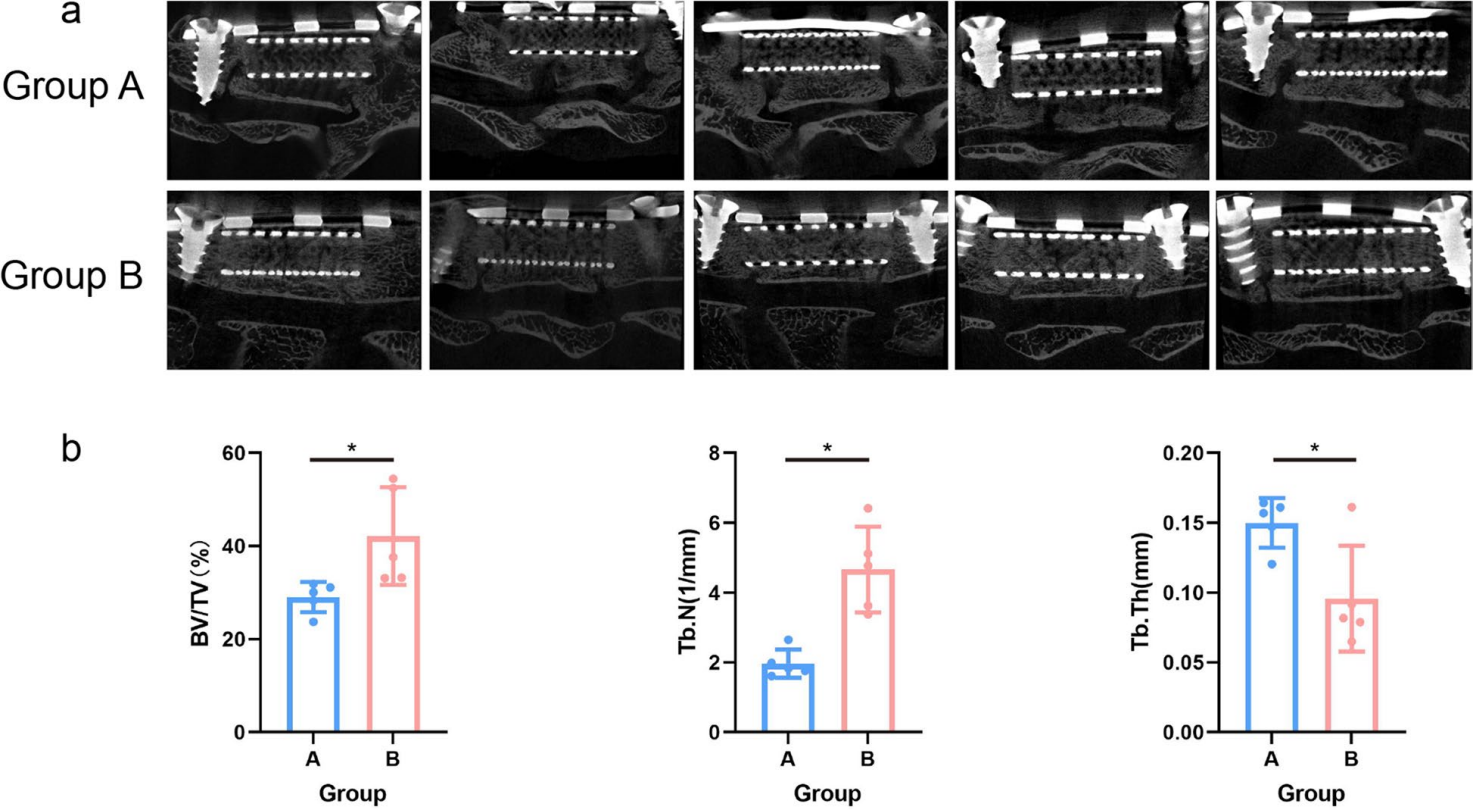
Micro-CT and quantitative analysis between the two groups. **a** The sagittal plane of the specimens, there were still cracks at the junction of titanium cage and endplate in some specimens at 4 weeks (Group A), there were no cracks detected at 8 weeks (Group B), and regular trabecular structure was observed. **b** The BV/TV, Tb.N, and Tb.Th in groups A and B. *, *P* < 0.05

The interbody space, where bony bridging was observed in both upper and lower levels, was deemed fused. Fusion was observed in two specimens of group A, while bony bridge formation was observed at only one interface in the other three specimens. Fusion occurred in all five specimens of group B (Fig. 5a). Fusion rates in groups A and B were 40% (2/5) and 100% (5/5), respectively, the difference being statistically significant (Table 2). Group B had significantly greater BV/TV (B: 42.20 ± 10.50 vs. A: 29.02 ± 3.25, *P* = 0.027) and Tb.N (B:4.66 ± 1.23 vs. A: 1.97 ± 0.40, *P* = 0.02) than group A (Fig. 5b), while Tb.Th was significantly lower in group B than A (B: 0.10 ± 0.04 vs. A: 0.15 ± 0.02, *P* = 0.019; Fig. 5b).

**Table 2:** Fusion rate of two groups

	Group		Sum	*P* value
	A	B		
Fused	2	5	7	0.038
Unfused	3	0	3	

### Histological result

**Fig. 6:**
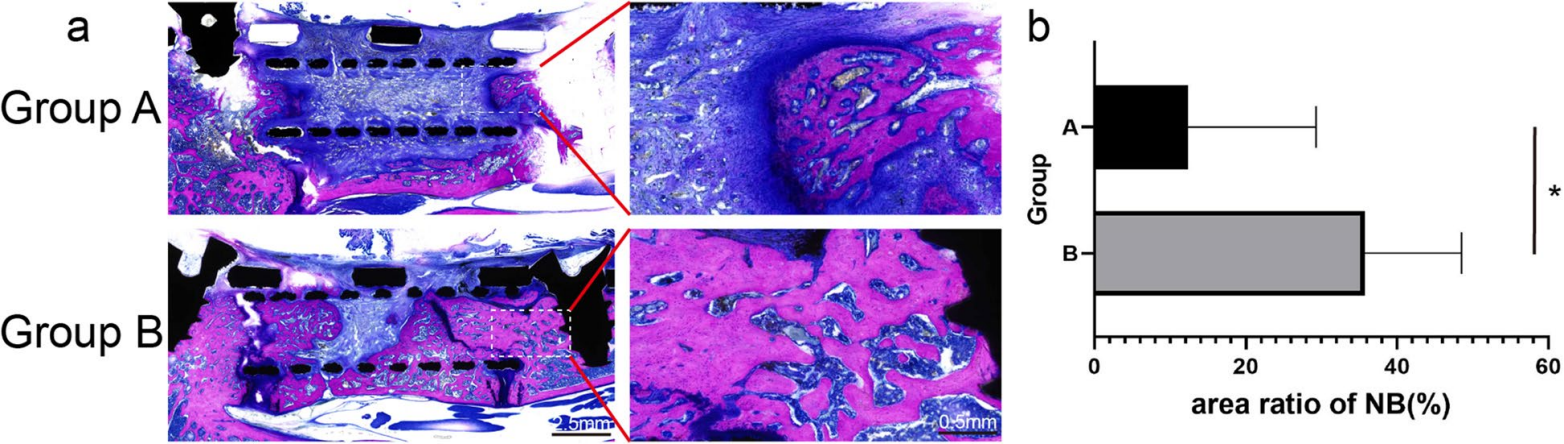
Methylene blue acid fuchsin staining and quantitative analysis between the two groups. **a** The photomicrographs show representative histological images of groups A and B, red for newly formed bone, pale blue for fibrous connective tissues, and dark blue for cartilaginous tissue. **b** The area ratio of NB of groups **A** and **B**. *, *P* < 0.05

In group A, little new osseous tissue (red area) was observed at the cephalad and caudal interbody spaces, and the space within the titanium cage was filled with a large number of fibrous connective tissues (pale blue area) and small amounts of cartilaginous tissue (dark blue area). Whereas in group B, the titanium cage and the two interbody spaces were filled with new osseous tissue, and fibrous tissue accounted for only a small proportion (Fig. 6a). The area ratio of newly formed bone (NB) in group A was lower than group B (A: 12.36 ± 16.93 vs. B: 35.72 ± 12.80, *P* = 0.039; Fig. 6b).

## Discussion

Intervertebral fusion models are predominantly performed on the cervical and lumbar spine in large animals, but in the rat interbody fusion model, an intertransverse process fusion model is more popular than the coccygeal interbody fusion model [19–22]. Xi Liang et al. used a goat cervical fusion model and found that the porous n-HA/PA66 strut offered the potential for cervical reconstruction after corpectomy [19]. Lovorka Grgurevic et al. constructed a sheep spine fusion model via anterior lumbar interbody fusion (ALIF) and posterolateral lumbar fusion (PLF) [20]. Jason R. Kang et al. used a rat intertransverse process fusion model to prove that varenicline had no adverse effect on spinal fusion [21]. Yu Cheng Yeh et al. characterized a coccygeal interbody fusion model and suggested it is an efficient model for future material and mechanical testing [22]. For research using animal models, larger animal models incur higher experimental costs. However, the existing rat model cannot fully simulate clinical surgery for interbody fusion. Therefore, we constructed a novel rat intervertebral fusion model based on anterior lumbar corpectomy and fusion. In this study, we developed an internal fixation for rat spinal fusion on account of directly measured L5 geometry. Bony fusion was observed at both four and 8 weeks post-surgery, with improved fusion rates at 8 weeks.

To our knowledge, there is no rat spinal fusion model constructed by anterior lumbar interbody fusion (ALIF). The surgical method is critical to this study and has a theoretical foundation in the research of Marc Antoine Rousseau et al., who reported the ventral approach to the lumbar spine of SD Rats [17]. The main points of this technique include: 1) The intestines and organs should be carefully protected during exposure by covering with wet gauze until fixation is complete; 2) The surgeon should be familiar with the anatomical structure of SD rats, particularly anatomical markers such as the posterior vena cava and ilio-lumbar vessels, which are key to locating the ventral aspect of the spine; 3) Due to the neurovascular plexus at L4 and the shielding effect of the pelvis at L6, the L5 vertebrae was selected to undergo corpectomy. A metal marker should be used for locating the L5 vertebrae via X-ray before corpectomy; 4) When revealing the ventral aspect of L5, the vertebral vessel across the middle of the L5 body can be seen (Fig. 3b). Thermocoagulation of the vertebral vessel ensures a clear surgical field. 5) Stringent aseptic measures should be adopted during model construction. To keep the incision clean and closed, skin interrupted sutures should be performed more intensive and every rat should be isolated in individual cages.

The presented model used the existing surgical approach and procedure for operation. The surgical approach was reported detailedly in Marc Antoine Rousseau’s study [17], and the key points of surgical procedure were described above. During the process of model establishment, we found that the ventral approach was feasible and with the assistance of microscope and microinstruments, the internal fixation could be implanted smoothly. It should be noted that basic surgery skill and asepsis were necessary for the surgical procedure. Additionally, titanium cages were yielded from the available 3D-Printer and based on the metrical data of L5. Collectively, The technique we used for model construction was feasible and reproducible.

Micro-CT is an emerging non-destructive imaging method that has become an essential assessment tool in animal spinal fusion research [23]. Spatial changes in bony architecture can be observed, and the amount of newly formed bone tissue can be calculated by Micro-CT, which enables comparisons between different interventions. Micro-CT has detected increases in bone mineral density (BMD), percent bone volume (BV/TV), trabecular thickness (Tb.Th), trabecular number (Tb.N), and decreases in trabecular separation (Tb.Sp) in a series of studies promoting spinal fusion [24–27]. In this study, BV/TV and Tb.N were greater in group B compared to group A, which is in line with previous research [24]. However, Tb.Th was lower in group B. This observation might be related to the early shaping processes of regenerated osseous tissue, which still needs further study. According to the Micro-CT images, we found that bone imaging within the titanium cage was more obvious in group B and that the forming trabecular architecture at the interfaces in group B (Fig. 5a). Overall, this study revealed that intervertebral fusion became more apparent as time progressed.

Histological examination is considered the gold standard method for assessing bone microstructure [28]. In this process, a section of bone tissue is stained with hematoxylin and eosin (H&E), Masson’s trichrome (MT), and methylene blue-acid fuchsin (MB/AF). Staining with H&E can easily identify calcium deposits, but cannot clearly differentiate osteoid and osseous tissues [29]. As a special staining technique, MT stain has been routinely used, although scanty bone tissue might be lost in the background of collagen [30]. It has been reported that MB/AF stain is a better way to differentiate bone and stromal tissues [31]. In this study, bone sections were stained with MB/AF. Four weeks post-surgery, the contents in the titanium cage was predominantly fibrous connective tissue. Newly formed bone tissue could be seen at both rostral and caudal ends of the cage, accompanied by a small cartilage formation. Eight weeks post-surgery, there was a mass of newly formed bone tissue in the titanium cage with less cartilage and fibrous connective tissue compared to the four-week post-surgery images. Quantitative analysis suggested the area ratio of newly formed bone of group B (8 weeks) was significantly greater than in group A (4 weeks). As such, the histological results confirmed that intervertebral fusion occurred in this animal model.

As trabecular bone grows along the direction of mechanical loading [32], a limitation of this study is the different spinal mechanics between rats and humans. Rats are quadrupedal animals with the spine parallel to the ground, while humans are bipedal with the spine perpendicular to the ground. Therefore, the human spinal mechanical environment cannot be directly simulated in the rat model. Future research could explore the effect of placing water and food at height to induce the rats to rise on their hind legs to obtain a similar mechanical stimulus.

## Conclusions

We developed a novel rat intervertebral fusion model based on anterior lumbar corpectomy and fusion (ALCF). The feasibility and efficacy of the model were verified by Micro-CT assessment and histological evaluation. This model may provide a new choice for fundamental research using animal models for spinal fusion.

## Data Availability

The datasets used and/or analyzed during the current study are not publicly available due to feasibility but are available from the corresponding author on reasonable request.

## Abbreviations

Micro-CT: Micro-computed tomography
ACLF: Anterior lumbar corpectomy and fusion
AP: Anteroposterior
BV: Bone volume
TV: Tissue volume
Tb.N: Trabecular bone number
Tb.Th: Trabecular bone thickness
MB/AF: Methylene blue-acid fuchsin
NB: Newly formed bone
